# Oral infection by the Human Papilloma Virus in women with cervical lesions at a prison in São Paulo, Brazil

**DOI:** 10.1590/S1808-86942012000200011

**Published:** 2015-10-20

**Authors:** Marco Antonio Zonta, Jussimara Monteiro, Gildo Santos, Antonio Carlos Campos Pignatari

**Affiliations:** a MSc in Clinical Tests – University of Santo Amaro (PhD Student of Infectology – Federal University of São Paulo – Unifesp); b PhD in Infectology – Federal University of São Paulo – Unifesp (Researcher of Infectology – Unifesp); c PhD in Infectology – Federal University of São Paulo – Unifesp (Head of the Molecular Biology Department of the Afp Lab); d PhD in Infectology – Federal University of São Paulo – Unifesp (Full Professor of Infectology – Unifesp). Universidade Federal de São Paulo – Departamento de Infectologia/Laboratório Especial de Microbiologia Clínica

**Keywords:** DNA probes, HPV, polymorphism, restriction fragment length, vaginal smears

## Abstract

Carcinoma of the head and neck is the 6^th^ cause of death by cancer in the world. In recent decades the human papillomavirus (HPV) has been implicated in the etiology of this disease

**Objective:**

To characterize the types of HPV detected in the oral mucosa in women with cytological abnormalities suggesting intraepithelial squamous lesions in the uterine cervix.

**Methods:**

four-hundred-nine cervical-vaginal and oral pap-smears of women interned in a Female Prison in São Paulo were examined. The relationship between cervical and oral lesion was analyzed by PCR/RFLP and DNA sequencing.

**Results:**

Of 27 (6.67%) specimens showing cervical cytological abnormalities suggesting LSIL and HSIL, 22 (81.48%) had oncogenic high-risk HPV infection, of which HPV 59 was the most prevalent. Three (11.1%) samples showed cytological changes suggesting mild dysplasia in the oral cavity.

**Conclusion:**

Our study suggests an association between carcinoma of the oral cavity and HPV infection, regardless of the virus type.

## INTRODUCTION

Head and neck cancer is the 5^th^ largest cause of cancer and the 6th largest cause of death by cancer in the world^1^. The largest incidence of this disease stems from the squamous cells of the upper digestive tract epithelium (oral cavity, pharynx and larynx). The main risk factors associated with the origin of this cancer are: smoking and alcoholism^2^.

Estimates for the year 2010 showed the occurrence of 14,120 new cases of oral cavity cancer, with a total of 6,214 cases of death caused by this disease, being the largest prevalence associated with males (10,330 cases)^3^.

In the last decades, infection by the Human Papilloma Virus (HPV) has been associated with the etiology of this disease, as it happens with uterine cervix cancer. It is believed that the oral infection may be acquired through oral or orogenital contact, or autoinfection. Sexual behavior has a direct relationship with the transmission mechanism of this viral infection, and the direct contact between the mucosae is the main route^4^, ^5^, ^6^, ^7^. Vertical transmission can also happen^8^, ^9^.

HPV is a DNA virus, epitheliotropic, non-enveloped, with a double strand circular genome, and about 8,000 pb, measuring 55 nm in diameter. Its genome is broken down into three regions: a long control region (LCR) and the Early (E) and Late (L) regions. Regions E and L code viral proteins, while the LCR is called non-coding region^10^, ^11^, ^12^.

This virus's oncogenic action is associated with the interaction of E6 and E7 oncoproteins, produced by the early genes E6 and E7, respectively. These proteins are involved in cell proliferation control, controlled by genes p53 and pRb, respectively, responsible for cell DNA control and correction, responsible for cell transformation and the malignant evolution of these lesions^13^, ^14^, ^15^.

HPV plays an important role in the oncogenesis of oral carcinoma, and its nomenclature is associated with its oncogenic potential. The pre-malignant lesions caused by the low-risk viruses are characterized by epithelial cells with nuclear atypias and cytoplasmic changes known as koilocyte, which have an enlarged nucleus, hyperchromia, perinuclear halo, binucleation or multinucleation and an increase in cytoplasm keratinization. These changes may also be found in high grade lesions, though less often^16^.

The low oncogenic risk viruses are: HPV-6, HPV-11, HPV-42, HPV-43, HPV-44, HPV-54, HPV-61, HPV-70, HPV-72 and the HPV-81, which cause benign lesions, known as oral warts, papilloma and white epithelium in the oral cavity. The high oncogenic risk viruses are: HPV-16, HPV-18, HPV-31, HPV-33, HPV-45, HPV-51, HPV-51, HPV-52, HPV-56, HPV-58, HPV-59, HPV-68, HPV-73 and the HPV-82. These are associated with atypical hyperplasia and oral carcinoma^17^, ^18^. In these cases, HPV-16 has been more frequently found^19^, ^20^, ^21^.

The goal of this study was to do a molecular characterization of HPV types diagnosed in the oral mucosa of the women who had cytological changes matching those of a low grade squamous lesion and a high grade one in the uterine cervix.

## MATERIALS AND METHODS

The present study is a cross-sectional contemporary cohort study, in which we studied 409 cervix-vaginal and oral cavity samples from women aged between 18 and 60 years of age, sexually active, inmates of the Women's Prison of the City of São Paulo, between February of 2006 and December of 2007. Initially, the women were submitted to an interview, aiming at obtaining clinical, sexual and social behavior information. The patients were instructed about and given the Informed Consent Form. This project was submitted to and approved by the Ethics in Research Committee of the Federal University of São Paulo (UNIFESP-CEP: 0369/06).

### Biological material gathering

The biological material was gathered in two stages: (i) cervix-vaginal material gathering; (ii) oral material gathering. We carried out two gatherings from each one of the cavities (cervix-vaginal and oral). Initially, the gathering was carried out by the conventional method (REF) and fixed in the slide using Citofix®. Following that, we carried out another gathering in the same region, using a brush with plastic bristles from the DNA-Citoloq® gathering kit, from Digene® – Brazil and it was preserved in a proprietary liquid medium, called UMC®. The same gathering procedure was utilized in order to gather material from the oral cavity^21^.

### Cytological analysis

The cytological samples gathered by the conventional method were submitted to Papanicolau dye and analyzed under light microscopy at the Cytopathology Department of the Biomedicine Lab in São Leopoldo/“IN CITO” by two clinical examiners. The cell changes were classified according to the Bethesda 2001 system.

### Molecular detection and characterization

The molecular characterization of the HPV types was carried out by the Polymerase Chain Reaction (PCR), RFLP and the Sequencing methods at the Molecular Biology Department of the Clinical Microbiology Special Lab (LEMC) of the Infectology Program of the UNIFESP and the Molecular Biology Lab from AFIP^3^. The HPV-DNA was detected by PCR. The HPV-DNA was extracted using the GFX® kit (GE Healthcare) used to extract the genomic DNA of epithelial cells. The HPV-DNA was amplified using the MY9 and MY11 universal primers, which amplify the large majority of HPV types. The product of such amplification was submitted to RFLP techniques, according to the protocol proposed by Bernard et al.^22^ and sequenced by the *Big Dye Terminator Cycle Sequencing* kit (Applied Biosystems, Foster City, CA). We used the ABI PRISM 310, *Genetic Analyzer* (Applied Biosystems, Perkin Elmer, California). The DNA sequences obtained were analyzed using the *Lasergene Software Package* (DNASTAR, Madison, WI) and, following that, they were submitted to comparison with the genetic data base^23^, ^24^.

## RESULTS

### Cytological analysis

Of the 409 cervix-vaginal samples gathered and assessed, 27 (6.67%) showed lesions in the uterine cervix, according to the cyto-pathology report. Among the 27 women who had lesions in the uterine cervix, three (11.11%) showed cell changes associated with mild dysplasia in the oral cavity, with mild dyskariosis in mature squamous cells, and 24 (88.89) showed reactive changes associated with inflammation, occasionally associated with infectious agents (Table 1). Only the 27 women who had pre-malignant and malignant lesions of the uterine cervix were assessed as to the presence of HPV in their oral cavities.

**Table 1:** Comparative table between the cervix-vaginal and oral samples infected by HPV.

	Uterine Cervix			Oropharynx	
N	Cytology	PCR/RFLP	Cytology	Sequencing	% Similarity (GeneBank)
1	HSIL – NIC II	HPV – 18	Negative	Negative	Not applicable
2	Inflammation/AGUS	NEG	Negative	Negative	Not applicable
3	ASC-US	HPV-6b	Negative	Negative	Not applicable
4	LSIL	HPV – 6b	Negative	HPV-59	(90%)
5	ASC-US	Negative	Negative	HPV-39	(98%)
6	LSIL	HPV – 61	Mild dysplasia	HPV-59	(88%)
7	ASC-US	Negative	Negative	HPV-59	(93%)
8	LSIL	HPV – 6b	Negative	HPV-59	(88%)
9	LSIL	HPV – 6 b	Negative	NEG	Not applicable
10	LSIL	HPV – 39	Negative	HPV-39	(97%)
11	LSIL	HPV – 34	Negative	HPV-59	(94%)
12	LSIL	HPV – 31	Negative	HPV-59	(92%)
13	LSIL	HPV – 6b	Negative	HPV-59	(89%)
14	LSIL	Negative	Negative	HPV59	(91%)
15	LSIL	HPV – 16	Negative	HPV59	(90%)
16	LSIL	HPV – 6b	Negative	HPV59	(93%)
17	LSIL	HPV – 6b	Mild dysplasia	HPV-16/59	(85/82%)
18	LSIL	HPV – 6b	Negative	HPV-58	(96%)
19	ASC-US	Negative	Negative	HPV-58	(99%)
20	ASC-US	Negative	Negative	HPV-58	(89%)
21	LSIL	HPV – 16	Negative	HPV-58	(98%)
22	HSIL	HPV – 16	Negative	HPV39	(94%)
23	LSIL	HPV – 6b	Negative	HPV-16	(82%)
24	LSIL	HPV – 16	Negative	HPV-58	(94%)
25	LSIL	HPV – 6b	Mild dysplasia	HPV-16	(91%)
26	HSIL/NIC III	HPV – 33	Negative	HPV-16/18	(90%)
27	Invasive CA	HPV – 18	Negative	undefined	Not applicable

LSIL: (Low-grade squamous intraepithelial lesion); ASC-US: (Squamous atypia of undetermined significance); Invasive CA: (Invasive squamous carcinoma).

### Molecular analysis

The 27 cervix-vaginal and oral cavity samples with cytology report suggestive of viral infection were confirmed as to the presence of HPV-DNA by PCR and characterized as to the type of HPV by the RFLP and sequencing methods.

Of the three samples which showed cell changes associated with mild oral cavity dysplasia, all (100%) had HPV-DNA, and one (33.33%) showed concurrent infections by HPV-16 and HPV-59; and these were the most prevalent types in the pre-malignant lesions.

Among the 27 oral and cervix samples submitted to molecular characterization, 18 (66.66%) had concurrent infection in both regions; nonetheless, only one sample (3.70%) had concurrent infections by the same type of virus, HPV-39 (Table 1).

Among the 27 samples gathered from the oral mucosa, 23 (85.18%) had HPV infection, 22 (81.48%) of those were caused by high oncogenic risk HPV, one sample (3.70%) was positive concerning the presence of HPV-DNA; nonetheless, it was not possible to characterize it in a molecular level. The largest prevalence of infection in women aged between 26 and 35 years of age (44.44%) and the most prevalent type of HPV characterized was HPV59, in 11 (40.75%) oral samples. Four (14.82%) samples did not have HPV infection. Two (7.41%) samples had two different types of HPV, one sample had HPV-16 and HPV-18 and another sample had HPV-16 and HPV-59 (Table 2).

**Table 2:** Prevalence of positive oral cavity samples for HPV–DNA identified by the sequencing method and classified by age range.

Age range (years)	HPV (+)	HPV(-)	Total
18 to 25	4 (14.82%)	0(0%)	5 (18.52%)
26 to 35	13 (48.14%)	3 (11.12)	15 (55.56)
36 to 45	6 (22.22)	0	6 (6.22)
46 to 55	0	1 (3.70%)	1 (3.70%)

Total	23 (81.48%)	4(14.82%)	27 (100%)

When we consider the sexual behavior of the 27 inmates who had a positive result for oral cavity HPV, it was possible to notice that of the 27 positive samples for HPV, 18 (66.7%) samples, in which HPV infection was detected, were from women with heterosexual behavior. Three (11.11%) positive samples were identified in women with bisexual behavior. Two (7.41%) samples came from homosexual women (Table 3).

**Table 3:** Positiveness rate for HPV characterized by the molecular sequencing in the samples of women classified according to sexual behavior.

Sexual orientation	HPV (+)	HPV(-)	Total
Heterosexual	18(66.67%)	3 (11.11%)	21 (77.78%)
Bisexual	3 (11.11%)	0 (0%)	3 (11.11%)
Homosexual	2 (7.41%)	1 (3.70%)	3 (11.11%)

Total	23 (85.18%)	4 (14.82%)	27(100%)

Among the 27 samples gathered from the oral cavity, 11 (40.75%) had a greater prevalence of HPV-59, two (7.40%) samples had HPV-16, and type 16 was seen in two more cases of concurrent infections. One sample (3.70%) had HPV-18, concurrent HPV-16, three (11.11%) samples had HPV-39, five (18.53%) samples had HPV-58; and HPV–59 was the most prevalent (Table 4).

**Table 4:** Prevalence of HPV types in oral samples gathered at the women's prison of the city of São Paulo.

Type of HPV	Samples	Percentage (%)
Negative	4	14.81
HPV 16*	2*	7.40
HPV 18	1	3.70
HPV 39	3	11.11
HPV 58	5	18.53
HPV 59	11	40.75
Undefined	1	3.70

Total	27	100

* HPV 16 was found in two isolated samples and also in two samples with simultaneous infections.

Among the 27 oral samples from the molecularly characterized women, 18 (66.67%) reported smoking and nine (33.33%) said they did not smoke. Such habit was seen more frequently among women aged between 26 and 35 years of age – 11 (40.74%) cases (Table 5).

**Table 5:** The table shows the occurrence rate of women smokers per age range.

SMOKER				
	YES		NO	
AGE	GENERAL	%	GENERAL	%
18–25	3	11.11%	1	3.70%
26–35	11	40.74%	4	14.81%
36–45	4	14.81%	2	7.41%
46–55	0	0.00%	2	7.41%

Total	18	66.66%	9	33.33%

Among the 18 smokers, 16 (59.26%) women were infected by HPB and two (7.40%) did not have HPV-DNA. Among the non-smoking women, seven (25.95%) had oral HPV infection and two (7.40%) of these women did not have oral viral infection (Table 6).

**Table 6:** This table shows the occurrence of HPV infection and pre-malignant lesions in women smokers and non-smokers from the women's prison of the city of São Paulo.

	Samples	HPV +	HPV -	MILD DYSPLASIA
Smokers	18 (66.66%)	16 (59.26%)	2 (7.40%)	2 (7.40%)
Non-smokers	9 (33.34%)	7 (25.93%)	2 (7.40%)	1 (3.70%)

Total	27 (100%)			

### DISCUSSION

The relationship between HPV infection and lesions on the oral mucosa and oropharynx still is controversial. Numerous studies showed the HPV in the intact oral mucosa, and infected by the pre-malignant and malignant lesions; the tonsil area seems to be the area of the greatest penetration and cell transformation induced by the HPV. More sensitive molecular techniques, such as molecular characterization by sequencing, enable a better quality in the identification of the viral type in its infection site. Sexual behavior, associated or not to risk factors such as cigarette smoking and alcohol increase virus penetration in the oral mucosa, thus increasing the likelihood of pre-malignant and malignant lesions.

Studies showed the association between this infection and oral lesions and oral carcinoma, varying between 25% and 80% of high risk HPV prevalence in samples gathered from oral cavity lavage or brushed^25^.

Our study showed 81.48% of HPV in the oral mucosa of women inmates of the women's prison of the city of São Paulo who had concurrent infections and lesions in the uterine cervix, suggesting an internal transmission.

Rintala et al.^26^ reported 42% of HPV oral infection in the mucosae of children, having a 10% persistence within a 3-year interval. The correlation with the genital infection was 36%. Our study suggested a good correlation among the women who had uterine cervix infection; nonetheless, the similarity between the viral types in the two sites was low, and only one patient had the same type of HPV in the oral cavity and uterine cervix, which suggested a low frequency of autoinfection. Saini et al.^27^ reported the occurrence of 5.71% of concurrent infection by the HPV in women who had uterine cervix cancer.

Beaudenon et al.^28^ had associated the occurrence of multiple infections by HPV types 13 and 32 in cases of focal epithelial hyperplasia in the oral cavity.

Our study found three oral samples which had pre-neoplastic cell changes, mild dysplasia and also infection by high-oncogenic risk HPV, and in one of them had concurrent infections by HPV types 16 and 59.

Insofar as the age range is concerned, we found a higher frequency of HPV in the oral cavities of women between 20 and 30 years of age, mean age of 25 years, encompassing 40.74% of infection cases. Smith et al.^29^ found 47.10% of HPV infection in women with the mean age of 24 years.

The study showed a greater prevalence of HPV-59, and in two cases there was oral dysplasia and one case of multiple infections associated with the HPV 16. Kreimer et al.^30^ found a greater prevalence of HPV-16 in samples obtained from the oral cavity lavage in HIV-positive patients; HPV 58 and 59 were found in a lesser frequency. Hanson et al.^31^ found HPV-16 and HPV-59 infections in samples obtained from the oral cavity lavage of patients with oral and oropharynx cancer in Sweden.

Hennessey et al.^32^ reported the presence of infections by HPV-58 and 59 in pre-malignant and malignant lesions in the oral cavity and suggested that these types of viruses have a high oncogenic potential for the development of neoplasia in the oropharynx mucosa.

The greater rate of HPV oral infection happened to heterosexual women, showing, in this case, that the deviation in behavior does not have any direct relation to the viral transmission mechanism. Our results corroborate the findings from D'souza *et* al.^33^, who found a greater occurrence of HPV oral infection in heterosexual women; nonetheless, they found a low prevalence of infections in smoking women. Xavier et al.^34^ found a greater occurrence of HPV oral infection in heterosexual and monogamist men; nonetheless, the prevalence of oral infection was found in one case only.

During a fair amount of time, cigarette smoking and alcohol ingestion were considered the main cofactors in the occurrence of oral cavity and oropharynx lesions. Today, it is believed that the HPV plays an important role in the development of these lesions. Our study showed that 66.66% of the women, prison inmates, were smokers; among them, 59.26% were infected by the high risk HPV. Studies have shown an association between 0 and 55% of HPV infection in smokers. It is believed that this risk factor causes sequential repairs to the oral mucosa, making it more susceptible for viral infection. Chen *et al.*^35^, studied tissues through *in situ* hybridization, and found 78% of high risk HPV infection in smokers with squamous papilloma.

Thus, following up the inmates of the women's prison of the city of São Paulo by means of preventive gynecological exams and clinical and molecular evaluation of the oral cavity may clarify the possible relationship between HPV infection and oral cavity lesions in women who have simultaneous infection of the female genital tract.

## CONCLUSION

According to our study, we may suggest the existence of an association between the development of oral cavity lesions and HPV infection; however, it did not show a direct association between the types of viruses present in the uterine cervix and in the oral cavity, and still a weak association with the sexual behavior of the inmates of the women's prison of the city of São Paulo.
